# Innovative Programmable Bio-Nano-Chip Digitizes Biology Using Sensors That Learn Bridging Biomarker Discovery and Clinical Implementation

**DOI:** 10.3389/fpubh.2017.00110

**Published:** 2017-05-22

**Authors:** Nicolaos J. Christodoulides, Michael P. McRae, Timothy J. Abram, Glennon W. Simmons, John T. McDevitt

**Affiliations:** ^1^Department of Biomaterials, Bioengineering Institute, New York University College of Dentistry, New York, NY, USA; ^2^SensoDx, LLC, New York, NY, USA

**Keywords:** non-invasive sampling, biomarkers, medical microdevices, *in vitro* diagnostics, point of care

## Abstract

The lack of standard tools and methodologies and the absence of a streamlined multimarker approval process have hindered the translation rate of new biomarkers into clinical practice for a variety of diseases afflicting humankind. Advanced novel technologies with superior analytical performance and reduced reagent costs, like the programmable bio-nano-chip system featured in this article, have potential to change the delivery of healthcare. This universal platform system has the capacity to digitize biology, resulting in a sensor modality with a capacity to learn. With well-planned device design, development, and distribution plans, there is an opportunity to translate benchtop discoveries in the genomics, proteomics, metabolomics, and glycomics fields by transforming the information content of key biomarkers into actionable signatures that can empower physicians and patients for a better management of healthcare. While the process is complicated and will take some time, showcased here are three application areas for this flexible platform that combines biomarker content with minimally invasive or non-invasive sampling, such as brush biopsy for oral cancer risk assessment; serum, plasma, and small volumes of blood for the assessment of cardiac risk and wellness; and oral fluid sampling for drugs of abuse testing at the point of need.

## The Current Status

Despite the abundance of biomarker discovery data, presently absent are the tools to retrieve and the processes to translate these initial findings into clinically relevant settings.

It has been estimated that the regulatory approval rate of new protein analytes in the United States over the past 15 years has remained steady, averaging a low rate of 1.5 new proteins per year despite tens of thousands of scientific articles describing advances in cancer and cardiac biomarkers (1, 2). Unless Food and Drug Administration (FDA) approval is secured, these biomarker discoveries remain in research settings and never enter widespread US clinical practice. Part of the problem here is the fact that distinct phases of the process, such as biomarker discovery, validation, and clinical implementation, remain disjointed. In fact, in the majority of the cases, each phase is completed by different groups utilizing different instruments and, even, different “standards.”

Likewise, up to this juncture, there has been no effective vehicle or technology platform to validate biomarkers in large studies and to ultimately drive these biomarkers to FDA approval and clinical practice. Thus, most modern bioscience research efforts remain largely decoupled from real-world clinical practice, for both the biomarkers and the devices that measure them, despite huge investments in translational research programs.

The typical device development process, whether from academia, national labs, or the industrial sector, is a succession of lengthy steps that often happen in a linear, suboptimal, and disjointed manner. The process is lengthy and inefficient, draining precious resources and momentum out of venture capital and federal funding, alike.

A case in point for the dismal translation rate of new medical tests into real-world practice is extracted from an analysis by Schully and coworkers (3) of the fiscal year 2007 extramural grant portfolio of the National Cancer Institute as well as cancer genetic research articles published in 2007. The group classified both funded grants and publications as follows: T0 as discovery research; T1 as research to develop a candidate health application (e.g., device or therapy); T2 as research that evaluates a candidate application and develops evidence-based recommendations; T3 as research that assesses how to integrate an evidence-based recommendation into cancer care and prevention; and T4 as research that assesses health outcomes and population impact. They found that only 1.8% of the grant portfolio and 0.6% of the published literature were T2 research or beyond.

Similar problems exist for other major disease areas such as cardiovascular disease (CVD), autoimmune disease, and many more. Importantly, the translation of new medical tests is also burdened by the plethora of disparate disciplines in the lab-on-a-chip/point-of-care (POC)/medical microdevice communities. Here, critical to the ultimate goal is the bridge between bioscience and microfabrication, two areas that up to this juncture have remained largely separate.

**Figure 1:**
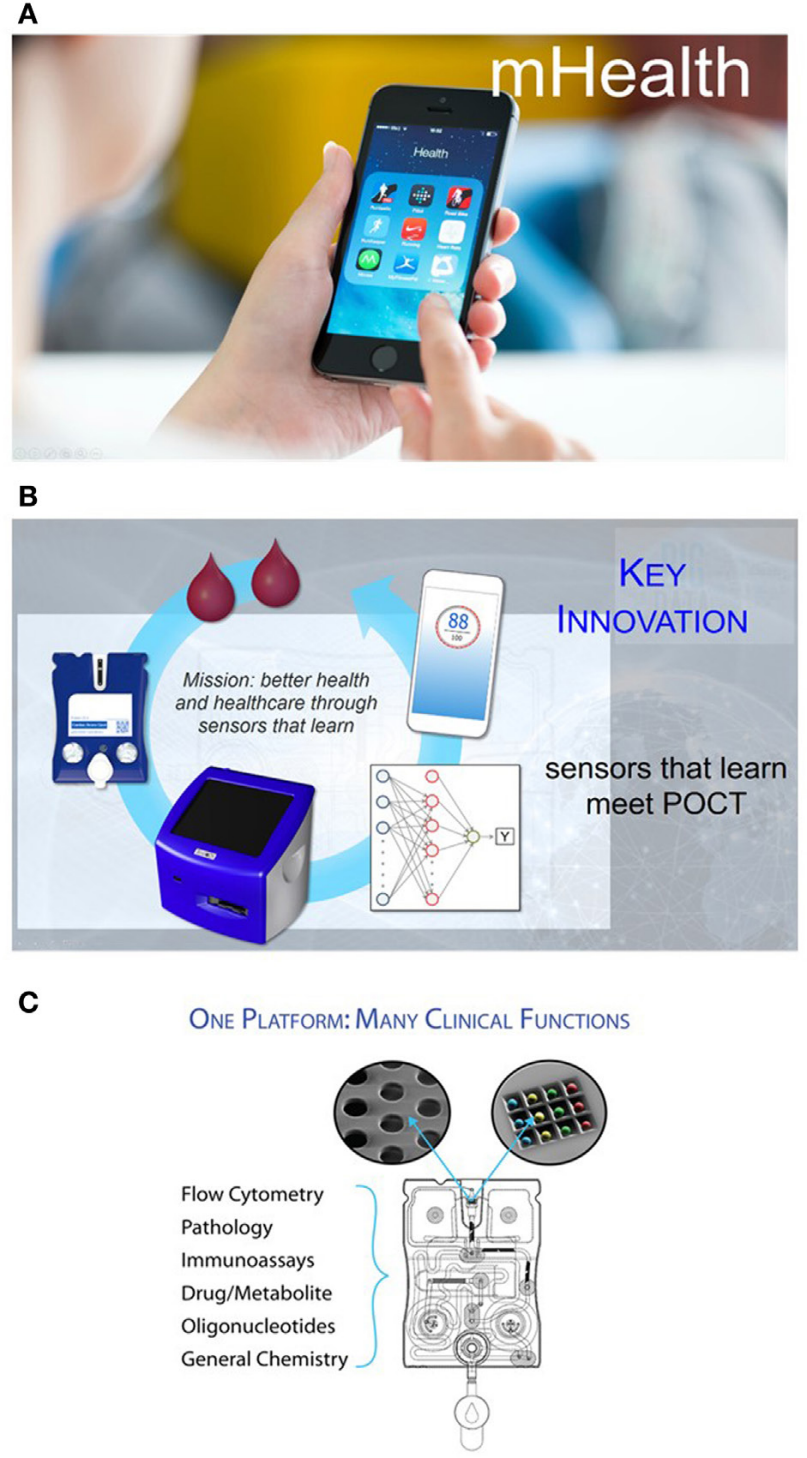
**(A)** Finding a way to provide patients and physicians the ability to cull biomarker information *via* diagnostic tests and then using the processing power and rich interface of smart phones opens the door to a more empowered patient and a more data savvy clinician. **(B)** The point-of-care (POC) programmable bio-nano-chip includes a universal analyzer with functional integrated mechanical/optical interfaces and flexible microchip architecture. An injection-molded, “credit-card”-sized cartridge encloses either a bead array or a membrane, where complex immunoassays or cellular analyses are performed, respectively. **(C)** Nanomaterials and microelectronics have been combined and adapted for the practical implementation of two classes of mini-sensors (membrane for cellular analyses and bead array for soluble target analyses) that read out with high-performance, yet affordable, imaging systems. These systems promise POC capabilities of tests traditionally completed by larger and more expensive systems confined in the lab, without sacrificing performance.

There is now a tremendous opportunity for new technologies to greatly enhance bench-to-bedside translation. Multiclass, multiplexed medical microdevices with enhanced throughput capabilities may help facilitate the translation of biomarkers into real-world practice (4). Integrating these new platforms with mobile health (mHealth) applications, an area that remained strikingly silent until today and lacked attention from the biomarker research community, may capitalize on the fact that an estimated 70% of all medical decisions regarding a patient’s diagnosis, treatment, hospital admission, and discharge are based on laboratory test results (Figure 1A). As health-care reform focuses on diagnosis and early intervention, timely and accurate lab quality mHealth results at the POC promise a higher quality of patient care and better management of downstream costs (5–7).

## Programmable Bio-Nano-Chip (p-BNC) Innovation

The use of new medical POC microdevices with minimally invasive or non-invasive sampling methods has the potential to expand *in vitro* diagnostics in traditional and non-traditional settings.

Pioneer work by Whitesides (8) in the basic sciences defined the ideal coatings, materials, and designs for the development of microfluidic channels and manipulations of biological fluids, while Quake’s group (9) advanced the “large-scale integration” of microfluidics, analogous to the electronics field. Other groups, such as those of Mirkin (10), Wang (11), and Heath (12), measured diverse sample types and created a variety of assembly types by using precious metal nanoparticles, nanowires, and magnetic techniques, respectively. Advancements by Sia (13) *via* microelectromechanical systems and by Singh (14) using chip-based separation and quantitation benefited integration of such systems. Others have extended their integrated approaches into the rapid, multiplexed detection of analytes, such as toxins and other biothreats (15), while Walt’s work (16) with electronic noses used arrays of optical fibers as the underlying infrastructure for biological sensing systems. Finally, researchers in the Toner group have explored a number of novel methods for the isolation and enumeration of lymphocytes, erythrocytes, and circulating tumor cells (17, 18).

These medical microdevices have recently reached the tipping point where they are starting to exceed the analytical performance of remote laboratory systems and do so with reduced cost. This exciting new trend overcomes previous limitations for POC technologies with respect to their higher cost and inferior performance, compared to macroscopic, laboratory-confined instrumentation. Likewise, these innovative POC technologies have the potential to serve as tools for more efficient translation of discovered biomarkers.

The p-BNC (Figure 1B) is one such platform technology that combines powerful machine-learning algorithms with unique chemsensing and biosensing capabilities (19–36).

The p-BNC includes a universal analyzer with functional integrated mechanical/optical interfaces and flexible microchip architecture. An injection-molded, “credit card”-sized cartridge encloses either a bead array or a membrane, where complex immunoassays or cellular analyses are performed, respectively. The cartridge is composed of a network of microfluidic components that ensure the complete transfer and processing of biological samples to provide quantitative information of the targeted biomarkers. The sample introduction requirements are consistent with the use of saliva, serum, plasma, and small volumes of blood for the assessment of cardiac risk and wellness or tissue brush samples that can be directly administered into the sample introduction port. Reagents are stored dry on a conjugate pad embedded within the biochip and are dissolved as needed through the release of prepacked buffer contained in biochip-integrated pouches. Here, all processing steps are conducted within the microfluidic network of the p-BNC *via* actuation inside the analyzer without human intervention. These features eliminate the need for external fluidics such as pumps, tubing, and connectors.

The p-BNC system combines and adapts nanomaterials and microelectronics to create two distinct classes of minisensors that read out with high-performance, yet affordable, imaging systems. Combined, the two p-BNC sensor ensembles form a modular platform compatible with possibly the largest and most diverse analyte portfolios available to date (Figure 1C). Importantly, the performance metrics of these miniaturized sensor systems have been shown to correlate closely with established macroscopic gold standard methods, making them suitable for use as subcomponents of highly functional detection systems for the analysis of complex fluid samples. These efforts remain unique in terms of functional p-BNC methods having a demonstrated capacity to meet or exceed the analytical characteristics (sensitivity, selectivity, assay variance, and limit of detection) of mature macroscopic instrumentation (19–36).

This platform is intended to be used outside of the traditional laboratory environments, such as in the ambulance, the emergency room, the intensive care unit, or cancer screening facilities at reduced cost with enhanced analytical and clinical performance. The rich data streams derived from this “Internet of Things”-enabled biosensing platform can be managed with a novel database, and mHealth tools can be integrated to enroll patients, manage and collect their data, and provide precision feedback to health-care providers and patients, alike.

## Platform Development and Validation

The p-BNC technology and methods were developed and validated in six major clinical studies funded by the National Institute of Dental and Craniofacial Research division of the National Institutes of Health, the Cancer Prevention Research Institute of Texas, and the United Kingdom’s Home Office Centre of Applied Science and Technology. These studies yielded innovations in application areas, including cardiac wellness, oral cancer screening, and drugs of abuse testing. These programs are described below.

## Cardiac Scorecard

**Figure 2:**
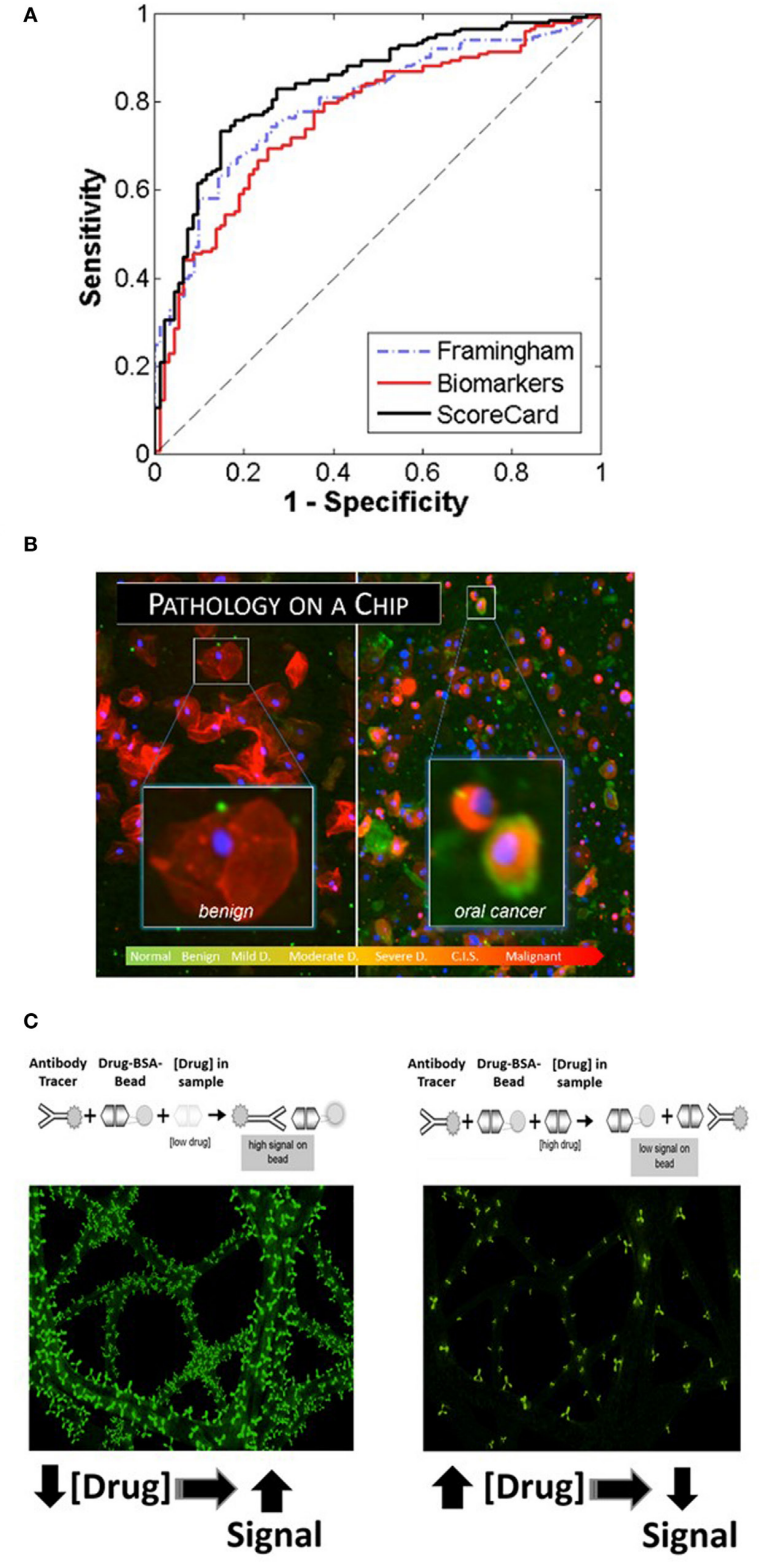
**Recent application areas of the programmable bio-nano-chip (p-BNC) include the Cardiac ScoreCard (A), the oral cancer test (B), and tests for drugs of abuse (C), all of which are conducted with minimally invasive or non-invasive sampling**. **(A)** Results of the cardiac wellness model showing receiver operating characteristic curves for the Framingham 10-year cardiovascular disease risk score (gold standard), biomarker model, and the ScoreCard. The Cardiac ScoreCard model shows superior performance for discriminating high- and low-risk subjects, demonstrating the importance of using both traditional risk factors and biomarker measurements in predicting cardiac wellness. **(B)** In the oral cancer area, the p-BNC platform was adapted to service a “cytology on a chip” application whereby cell and nuclear morphology along with cancer-associated biomarkers are measured using non-invasive brush biopsy sampling. **(C)** For drug testing, the bead-based p-BNC platform was programmed to host a fully quantitative, competitive type of immunoassay, whereby fluorescent signal on the bead was inversely proportional to the drug levels in the oral fluid sample processed.

Cardiovascular disease, including coronary artery disease, remains the leading cause of death and a major contributor to health-care costs worldwide. While it is highly desirable to have tools suitable for early detection for effective management of CVD, the modern cardiac diagnostics area tends to focus on late-stage disease syndromes like acute myocardial infarction (AMI) and heart failure (HF) (37, 38). Lacking today are tools to manage patients from holistic cardio perspective. The authors have developed recently the Cardiac ScoreCard test, a series of models based on a multivariate index assay with the potential to assist in the diagnosis and prognosis of multiple CVD progression levels (39, 40). The approach implements three lasso penalized logistic regression models in the areas of cardiac wellness, AMI, and HF. Validated through a major clinical study involving 1,050 subjects, the Cardiac ScoreCard test uses a panel of up to 16 biomarkers to yield a number between 0 and 100 that is indicative of overall cardiac wellness (Figure 2A). The score is literally interpreted as “the probability of being healthy,” as derived from a population of recruited healthy individuals and non-case chest pain patients. The Cardiac ScoreCard applies a time- and cost-effective approach in an attempt to develop predictive models for diagnosis and assessment of cardiac wellness, AMI, and HF. This expanded panel has redundancy that may eliminate blind spots in current diagnosis and prognosis and help establish useful baseline measurements for future reference that could assist in the development of more powerful learning algorithms.

## Oral Cancer

Oral squamous cell carcinoma (OSCC) is a significant health problem afflicting approximately 400,000 people globally each and every year. In the United States alone, greater than 45,000 new cases and nearly 8,650 deaths are estimated in 2015, representing approximately 4% of all cancers in men and 3% in women (41). The significant morbidity and mortality associated with OSCC is often attributed to the advanced disease stage of many OSCCs when the initial identification and biopsy are done. Despite advances in surgical procedures and treatment, the long-term prognosis for patients with OSCC remains poor with a 5-year survival rate at approximately 64%. This prognosis is among the lowest for all cancers. However, the survival rate increases dramatically to 83% when this abnormality is detected in its early stage. Unfortunately, only 32–47% of oral cancer cases are detected in the early stages. In addition to improving survival, early detection of oral cancers can identify the disease when less aggressive, non-disfiguring, less toxic, and less costly therapies are required, underscoring the need for new diagnostic methods that are able to identify early-stage disease. In the essential oral cancer area, the p-BNC platform was adapted to service a “cytology on a chip*”* application whereby cell and nuclear morphology along with expression of cancer-associated biomarkers are measured using non-invasive brush biopsy sampling (42–45). The 999-patient, 4-site international clinical trial, sponsored by the National Institutes of Health Grand Opportunity program, has led to the establishment of one of the largest oral cytology databases ever for potentially malignant oral lesions (PMOLs). Unlike existing adjunctive diagnostic aids, the new p-BNC data have fostered a robust capacity to assess in a quantitative manner the level of disease progression for PMOLs using a non-invasive sampling method combined with the automated chip-based sampling modality. This new approach exceeds in a major way the level of quantitation possible with existing adjunctive oral cancer diagnostic aids (45). From these careful studies of model performance, it is clear that the chip-based image approaches are effective for classifying patients in six diagnostic categories (Figure 2B). Sensitivity values in excess of 90% with specificity values around 75% have been achieved for this prospective trial exceeding capabilities of existing commercial approaches. Over 200 cellular features related to biomarker expression, nuclear parameters and cellular morphology were recorded per cell. By cataloging an average of 2,000 cells per patient, these efforts resulted in nearly 13 million indexed objects. Binary “low-risk”/“high-risk” models yielded AUC values of 0.88 and 0.84 for training and validation models, respectively, with an accompanying difference in sensitivity + specificity of 6.2%. In terms of accuracy, this model accurately predicted the correct diagnosis approximately 70% of the time, compared to the 69% initial agreement rate of the pool of expert pathologists. Key parameters identified in these models included cell circularity, Ki67 and EGFR expression, nuclear–cytoplasmic ratio, nuclear area, and cell area. This chip-based approach yields objective data that can be leveraged for diagnosis and management of patients with PMOL as well as uncovers new molecular-level insights behind cytological differences across the OED spectrum.

## Sensors for Drugs of Abuse

Current onsite detection methods for drugs of abuse involve invasive sampling of blood and urine specimens, or collection of oral fluid, followed by qualitative screening tests using immunochromatographic cartridges. The instrumentation used by remote laboratories to provide subsequent confirmation and quantitative assessment of a presumptive positive is expensive and decoupled from the initial sampling, making the current drug-screening program inefficient and costly. In contrast, the p-BNC assay methodology (applied recently for the detection of 12 drugs of abuse initially in spiked buffered samples and, ultimately, in non-invasive oral fluid specimen collected from consented volunteers) is easy to use, rapid, low cost, multiplexed, and fully quantitative (Figure 2C) (46, 47).

Customized macros developed and optimized for the automated analysis of drug-specific bead-based assays serve to determine the exact bead location on the chip, followed by their respective bead-specific assignments and to extract bead data using “regional pixel extraction analysis” strategies that are automatically applied for the generation of dose response curves and used for the measurement of the various drug concentrations in unknown samples. Analysis of each bead within the group, for each of the drug tests reported here, at each exposure time was initiated within the macro through line profile and circular area of interest routines for data extraction (46, 47).

Furthermore, the system’s superior analytical performance allows visibility to time course of select drug and metabolite profiles in oral fluids that, when combined with concentration measurements from this and prior impairment studies, reveals additional information about drug-induced impairment. This chip-based p-BNC detection modality promises to serve as a valuable tool in law enforcement roadside drug testing and for a variety of other settings, including outpatient and inpatient drug rehabilitation centers, emergency rooms, prisons, schools, and the workplace.

Simple to use, rapid, minimally invasive or non-invasive, accurate, and sensitive technologies, such as the p-BNC, that offer advanced screening, diagnosis, and monitoring of diseases in a variety of settings (e.g., physicians’ and dentists’ offices, pharmacies, and at home) promise to overcome major past limitations for POC technologies, such as cost and lack of sensitivity.

By integrating experts in clinical unmet needs and market assessment, clinical research, device development and testing, regulatory process and commercialization, and outreach for education and training, there is promise to improve success rates for the translation of medical devices and of the biomarker tests for which these technologies host.

## Author Contributions

JM: principal investigator, inventor of programmable bio-nano-chip technology. NC: senior scientist in McDevitt lab, director of assay development, and drug study project manager. MM: developed CardiacScoreCard and latest iteration of portable analyzer and cardiac study project manager. TA: scientist at SensoDx and oral cancer study manager. GS: p-BNC cartridge development engineering lead.

## Conflict of Interest Statement

JM is one of the founders of SensoDx and serves as the chief scientific officer for the same. The other authors declare no conflict of interest.
